# Risk Acceptance in Multiple Sclerosis Patients on Natalizumab Treatment

**DOI:** 10.1371/journal.pone.0082796

**Published:** 2013-12-10

**Authors:** Carmen Tur, Mar Tintoré, Ángela Vidal-Jordana, Denis Bichuetti, Pablo Nieto González, María Jesús Arévalo, Georgina Arrambide, Elisenda Anglada, Ingrid Galán, Joaquín Castilló, Carlos Nos, Jordi Río, María Isabel Martín, Manuel Comabella, Jaume Sastre-Garriga, Xavier Montalban

**Affiliations:** 1 Multiple Sclerosis Centre of Catalonia (Cemcat), Department of Neurology-Neuroimmunology, Vall d’Hebron University Hospital, Barcelona, Spain; 2 Department of Neurology and Neurosurgery, Federal University of São Paulo, São Paulo, Brazil; 3 Department of Neurology, University Hospital Príncipe de Asturias, Alcalá de Henares, Madrid, Spain; Friedrich-Alexander University Erlangen, Germany

## Abstract

**Objective:**

We aimed to investigate the ability of natalizumab (NTZ)-treated patients to assume treatment-associated risks and the factors involved in such risk acceptance.

**Methods:**

From a total of 185 patients, 114 patients on NTZ as of July 2011 carried out a comprehensive survey. We obtained disease severity perception scores, personality traits’ scores, and risk-acceptance scores (RAS) so that higher RAS indicated higher risk acceptance. We recorded JC virus status (JCV+/-), prior immunosuppression, NTZ treatment duration, and clinical characteristics. NTZ patients were split into subgroups (A-E), depending on their individual PML risk. Some 22 MS patients on first-line drugs (DMD) acted as controls.

**Results:**

No differences between treatment groups were observed in disease severity perception and personality traits. RAS were higher in NTZ than in DMD patients (p<0.01). Perception of the own disease as a more severe condition tended to predict higher RAS (p=0.07). Higher neuroticism scores predicted higher RAS in the NTZ group as a whole (p=0.04), and in high PML-risk subgroups (A-B) (p=0.02). In low PML-risk subgroups (C-E), higher RAS were associated with a JCV+ status (p=0.01). Neither disability scores nor pre-treatment relapse rate predicted RAS in either group.

**Conclusions:**

Risk acceptance is a multifactorial phenomenon, which might be partly explained by an adaptive process, in light of the higher risk acceptance amongst NTZ-treated patients and, especially, amongst those who are JCV seropositive but still have low PML risk, but which seems also intimately related to personality traits.

## Introduction

Multiple sclerosis (MS), an inflammatory-demyelinating disease of the central nervous system (CNS), is the second most frequent cause of disability amongst young adults[1]. Over the last decade, an increasing number of new drugs have been tried in patients with RRMS, with encouraging results, showing greater efficacy than conventional first-line disease modifying drugs (DMD)[2-12]. Some of these new molecules, such as natalizumab (NTZ), fingolimod, and teriflunomide (in the US), are already available on the market for patients with RRMS[13], and some others will probably be available in the near future. Therefore, drastic changes in the therapeutic scenario are to be expected over the next few years, which may have an important impact on the natural history of the disease. However, these highly effective drugs are likely to entail some risk of potentially serious -although generally infrequent- adverse events that patients will have to assume if they want to benefit from them.

The efficacy of NTZ has been greatly demonstrated in the setting of clinical trials[2-5] and day-to-day clinical practice[14]. However, its use has been limited by the risk of progressive multifocal leukoencephalopathy (PML), an opportunistic infection caused by the John Cunningham virus (JCV), which may have fatal consequences in 20% of those affected, or lead to serious disability in 40% of survivors[15]. PML risk is associated to long NTZ treatment schemes, JCV seropositivity, and a past history of immunosuppressant (IS) drug treatment[15-18]. Up to now, more than 300 cases of PML have been diagnosed worldwide[13]. At present, due to the high efficacy of NTZ, numerous RRMS patients are reaching the timepoint of two years of treatment and need to face the decision whether to assume a higher PML risk or switch to other second-line drugs, whose efficacy has not been compared to NTZ, which could therefore mean a deterioration of their disease. Apart from that, given that the other second-line drugs can also entail a risk of secondary effects, it is likely that, as time goes by, clinicians encounter an increasing number of patients whose prognosis eventually depends on their ability to assume treatment-associated risks. Thus, it is of the highest importance to know the reasons that can lead to a given patient to decide whether to continue on a given treatment or not. 

In 2012, we reported that amongst the NTZ-treated patients who had the three PML risk factors, those with lower reductions in disability scores while on treatment were more likely to discontinue NTZ after a decision-making process[19]. We also found that the neurologist with whom treatment discontinuation had been discussed played a major role in the final decision. Nonetheless, we did not study the involvement of other factors such as personality traits or the perception of MS as a serious, disabling disease. 

The aim of the present study was to investigate the ability of MS patients to accept risks associated to treatments, and the factors involved in this risk acceptance. We focused on patients who were already receiving NTZ, as a paradigm of second-line treatment, and hypothesised that patients on NTZ, with an *a priori* more severe disease than patients on first-line disease modifying drugs (DMD), would be more prone to accept higher risks than DMD patients. We also hypothesised that the perception of MS as a more severe disease and the presence of greater disability scores would make patients more prone to accept higher risks associated to MS treatments. 

## Methods

### Participants

From a total of 185 MS patients[20-22] who had received at least one dose of NTZ in our centre, 141 were receiving this drug by July 2011. Of them, four patients refused to participate, one patient had a language barrier and we considered that his inclusion was not appropriate, and 22 patients were not invited for different reasons: pregnancy planning (five patients, all of them had just stopped treatment), participation in another study (five patients, who were about to start a clinical trial), and agenda issues (12 patients). Therefore, a total of 114 patients (81%) on NTZ treatment (that we called ‘NTZ group’) finally participated in this study. They were not different from those who did not participate (N=27), in terms of disease duration (t-test: p=0.558), age (t-test: p=0.978), treatment duration (t-test: p=0.259), and disability scores at NTZ onset (Mann-Whitney U test: p=0.201). All NTZ-treated patients received NTZ in the approved indication, that is, because they had suffered from disabling relapses despite being on first-line DMD. However, 15 out of the 114 NTZ-treated patients who participated in this study had an EDSS score at treatment onset greater than 5.5, which is the maximum EDSS score allowed to receive NTZ in our setting. In those cases, we had to ask for permission to the competent authorities of our hospital to be able to offer NTZ to these patients. 

Besides, a group of 22 patients on first-line DMD was also studied (‘DMD group’). They were consecutive patients who attended the Day Hospital of our centre, either to start on first-line DMD (10 patients) or to change from a first-line DMD to another first-line DMD (12 patients). 

### Clinical and demographical characteristics

Age, gender, disease duration, Expanded Disability Status Scale (EDSS)[23] scores at the time of the study, and the annualised relapse rates (ARR) during the year before starting on treatment, either on NTZ or first-line DMD, for the ‘NTZ group’ or the ‘DMD group’, respectively, were recorded. All relapses and EDSS scores over time were recorded using patients’ files, which could be either physical (they contained information up to 2009), or electronic (they contained information after 2009). We also recorded, in the ‘NTZ group’, variables associated with the individual risk of PML, such as doses of NTZ received, presence of anti-JCV antibodies, and past history of immunosuppression (IS). Thereafter, NTZ patients were split into five subgroups, depending on their individualised estimated PML risk: from A, with highest PML risk, to E, with lowest PML risk, according to current knowledge about natalizumab-associated PML as of July 2011[18] (Table **1**). 

**Table 1:** Distribution of NTZ-treated patients according to individualised estimated PML risks.

**PML risk group**	**JCV status**	**Number of NTZ infusions**	**Prior IS**	**Estimated PML risk^*a*^**	**Estimated PML risk^*b*^**	**Number of patients**
**A**	+	> 24	+	8.1/1000 (5.4–11.6)	11.1/1000 (8.3–14.5)	3
**B**	+	> 24	-	2.8/1000 (2.0–3.8)	4.6/1000 (3.7–5.6)	32
**C**	+	< 24	+	1.2/1000 (0.58–2.2)	1.6/1000 (0.91–2.6)	2
**D**	+	< 24	-	0.35/1000 (0.19–0.60)	0.56/1000 (0.36–0.83)	17
**E**	-	< 24 or > 24	+ or -	<0.11/1000 (0–0.59)	<0.09/1000 (0–0.48)	60
**A and B (together)**	+	> 24	+ or -	-	-	35
**C to E (together)**	+ or -	< 24 or > 24	+ or -	-	-	79

This table shows the distribution of patients according to their estimated PML risk. a: PML risks are expressed as mean (95% CI). according to the information provided by Sandrock et al. (Sandrock et al. P03.248. AAN 2011); b: risks are expressed as mean (95% CI). according to the information provided by Bloomgren et al.. N Engl J Med 2012. *Abbreviations*: JCV: JC virus; IS: immunosuppression.

### Assessment of the perception of MS severity

For this purpose we used a survey containing visual analogic scale (VAS) questions, whose possible values ranged from 0 to 10, as has been previously used[24]. Patients were asked to answer to what extent they thought MS was a severe disease (0 = MS is not at all a severe disease; 10 = MS is the most severe disease you can think of), in general (i.e. for MS patients as a whole) and in their particular case. We therefore obtained two severity perception scores, i.e. a general and an individual severity perception score, per patient.

### Assessment of personality traits

For this purpose, the NEO Five-Factor Inventory of personality traits was administered[25]. It explores five personality traits, namely neuroticism, extroversion, openness, agreeableness, and responsibility, by means of 60 questions (12 questions per personality trait) whose scores ranged from 0 to 4. Thereafter, averaged scores for each trait were obtained, according to official guidelines[25]. 

### Assessment of risk acceptance

Here, we presented five hypothetical therapeutic scenarios, which had five different associated risks of a serious secondary effect. Patients were asked to what extent they would like to continue receiving a given drug if the associated annualised risk of a serious secondary effect was 1/2,000,000 (very low risk [therapeutic] scenario), 1/600,000 (low risk scenario), 1/5,000 (intermediate risk scenario), 1/100 (high risk scenario), and 1/50 (very high risk scenario). Patients had to answer these questions using visual analogic scales (VAS), whose possible values ranged from 0 to 10 (0 = *I would not like to continue receiving this drug at all*; 10 = *I would like to continue receiving this drug without any doubt*). In order to help patients to understand the risks associated to each therapeutic scenario, we also presented five events totally unrelated to MS, whose associated risks or likelihoods were similar to those associated to the hypothetical therapeutic scenarios (Table **S1**). Therefore, we obtained risk-acceptance scores (RAS), one RAS for each therapeutic scenario, so that higher RAS indicated better acceptance of risk. 

Finally we averaged RAS for high and very high-risk scenarios, thus obtaining a variable called ‘averaged RAS’, which indicated the degree of acceptance of high and very high treatment-associated risks. 

### Statistical analysis

This study has a cross-sectional design. 

To assess differences between treatment groups (‘NTZ group’ and ‘DMD group’) in terms of clinical and demographical characteristics, general and individual severity perception scores, RAS and ‘averaged RAS’, we used t-tests, Mann-Whitney U tests, or chi-square tests, depending on the variable to be compared. To assess internal consistency of patients’ answers related to risk acceptance, paired t-tests were performed to investigate differences between very-high- and high-risk RAS, high- and medium-risk RAS, medium- and low-risk RAS, low- and very-low risk RAS. 

To investigate factors involved in risk acceptance, multiple linear regression analyses were performed so that the ‘averaged RAS’ was taken as the dependent variable, and the remaining variables, i.e. clinical and demographical data, general and particular severity perception scores, and personality traits’ scores were taken as independent variables. In order to give information about the strength of the association between the dependent and the independent variables, correlation coefficients obtained within the multiple regression analyses performed are also reported (when statistically significant). 

All analyses were performed with IBM® SPSS® Statistics version 20. Statistical significance was considered whenever p value was lower than 0.05. This study was approved by the Ethics Committee of the Vall d’Hebron Research Institute and by the Spanish Agency for Medicines and Health Products (*Agencia Española de Medicamentos y Productos Sanitarios* [AEMPS]). Participants provided their written informed consent to participate in this study. Both Ethics Committees approved this consent procedure.

## Results

### Clinical and demographical characteristics, disease severity perception, and personality traits

Clinical and demographical characteristics are shown in Table **2**. As expected, NTZ patients had longer disease durations (p<0.001), greater EDSS scores at the time of the study (p<0.001), and higher ARR over the year before starting on treatment (p=0.037) than DMD patients. No differences were observed between treatment groups in terms of age, gender distribution, or treatment duration. 

**Table 2:** Clinical and demographical characteristics, disease severity perception scores, and personality traits.

	**NTZ group N = 114**	**DMD group N = 22**	**p value**
**Clinical and demographical characteristics**			
Age (years)**^*a*^**	37.78 (8.12)	39.16 (11.11)	0.582**^*c*^**
Gender (males. %)	34 (30%)	6 (27%)	0.810**^*d*^**
Disease duration (years)**^*a*^**	12.92 (6.90)	5.21 (4.39)	**<0.001^*c*^**
EDSS scores**^*b*^**	3.75 (0-8)	2.00 (1-5)	**<0.001^*e*^**
ARR (over the year before starting on treatment)**^*a*^**	1.95 (0.95)	1.45 (1.05)	**0.037^*c*^**
Treatment duration (years)**^*a*^**	2.55 (1.74)	2.45 (3.09)	0.889**^*c*^**
**Disease severity perception scores (range: 0-10)^*a*^**			
In general	7.00 (1.97)	7.62 (1.75)	0.184**^*c*^**
In their particular case	5.77 (2.12)**^*f*^**	6.10 (1.79)**^*f*^**	0.507**^*c*^**
**Personality traits scores (range: 0-4)^*a*^**			
Neuroticism	1.77 (0.73)	1.66 (0.65)	0.536**^*c*^**
Extroversion	2.42 (0.69)	2.44 (0.49)	0.881**^*c*^**
Openness	2.29 (0.60)	2.15 (0.46)	0.307**^*c*^**
Agreeableness	2.60 (0.50)	2.61 (0.41)	0.962**^*c*^**
Responsibility	2.65 (0.56)	2.59 (0.48)	0.612**^*c*^**

**a**: mean (standard deviation); **b**: median (range); **c**: independent samples t-test (NTZ group vs. DMD group); **d**: Chi-square test (NTZ group vs. DMD group); **e**: Mann-Whitney U test (NTZ group vs. DMD group); **f**: to compare disease severity perception in their particular case vs. disease severity perception in general: related samples t-test (NTZ group: p<0.001; DMD group: p<0.001). Abbreviations: ARR: annualised relapse rate; DMD: disease modifying drugs; EDSS: expanded disability status scale; NTZ: natalizumab.

As regards the PML risk factors amongst NTZ-treated patients, 54 were JCV seropositive, and 60 JCV seronegative. Amongst those JCV seropositive patients, 35 had received more than 24 NTZ doses (> 2 years); amongst these, 3 had also received mitoxantrone in the past (group A) and 32 had not received any kind of IS (group B). The distribution of all NTZ-treated patients according to their individualised estimated PML risks is shown in Table **1**. 

In both treatment groups, patients considered that their own disease was significantly less severe than was MS in general (NTZ group: p<0.001; DMD group: p<0.001), but no significant differences were observed between treatment groups in any of the two severity perception scores (Table **2**). Finally, personality traits were similar between both treatment groups (Table **2**).

### Treatment-associated risk acceptance

NTZ patients showed significantly higher RAS than DMD patients, for all five hypothetical therapeutic scenarios that we presented (Table **3**), indicating a higher acceptance of treatment-associated risks. Furthermore, the higher the risk the hypothetical therapeutic scenario had, the lower the RAS was (Table **3**). 

**Table 3:** Treatment-associated risk-acceptance scores.

	**Risk-acceptance Scores (range: 0-10)^*a*^**		
**Therapeutic scenarios-associated risk**	**NTZ group**	**DMD group**	**p value^*b*^**
Very low (1(2.000.000)	8.85 (1.94)	7.50 (2.26)	**0.019**
Low (1/600.000)	8.49 (2.18)	6.32 (2.71)	**<0.001**
Medium (1/5.000)	7.47 (2.75)	4.76 (3.07)	**<0.001**
High (1/100)	4.29 (3.50)	2.43 (2.03)	**0.002**
Very high (1/50)	3.01 (3.36)	1.58 (1.83)	**0.008**
Average High and Very High**^*c*^**	3.66 (3.35)	2.00 (1.86)	**0.003**

a: mean (standard deviation); b: independent samples t-test; c: averaged Risk-Acceptance Scores for high and very high-risk scenarios (‘avRAS’).

Paired t-tests showed significant differences between very-high- and high-risk RAS (p<0.001 [for NTZ group]; p=0.002 [for DMD group]), high- and medium-risk RAS (p<0.001 [NTZ]; p=0.001 [DMD]), medium- and low-risk RAS (p<0.001 [NTZ]; p=0.008 [DMD]), and low- and very-low risk RAS (p=0.047 [NTZ]; p=0.002 [DMD]), indicating a good internal consistency of patients’ answers. 

### Factors involved in risk acceptance

When all patients were investigated as a whole (NTZ and DMD groups together), there was a trend towards a relationship between higher ‘averaged RAS’ and higher disease severity perception scores in each particular case (i.e. by the perception of having a more severe disease) (p=0.052). Similarly, there was a trend towards a relationship between higher ‘averaged RAS’ and higher neuroticism scores (p=0.089), after correcting for treatment group (Table **4**). 

**Table 4:** Factors involved in risk acceptance.

	**Population studied**			
	NTZ + DMD (all patients)**^*a*^**	NTZ (A-E [all] groups)	NTZ (A-B groups)	NTZ (C-E groups)
**Predictive variables**				
Age and gender	-	-	-	-
Disease duration	-	-	-	-
EDSS	-	-	-	-
ARR	-	-	-	-
Positive JCV-antibodies	-	-	-	p=0.015; CC=0.27
Prior IS	-	-	-	-
Disease severity perception scores, in general	-	-	-	-
Disease severity perception scores, in their particular case	p=0.052; CC=0.17	-	-	-
Personality traits (neuroticism scores)**^*b*^**	p=0.089; CC=0.15	p=0.042; CC=0.20	p=0.015; CC=0.42	-

**a**: Multiple linear regression analyses are adjusted for treatment group, given the strong association between treatment group and some clinical and demographical variables; **b**: Neuroticism was the only personality trait that was associated with risk acceptance; thus, higher neuroticism scores were related to higher risk acceptance in those groups that are shown in the table. Abbreviations: ARR: annualised relapse rate; CC: correlation coefficients obtained from the multiple linear regression analyses performed; DMD: disease modifying drugs; EDSS: Expanded Disability Status Scale scores; IS: immunosuppression; JCV: JC virus; NTZ: natalizumab.

**Figure 1:**
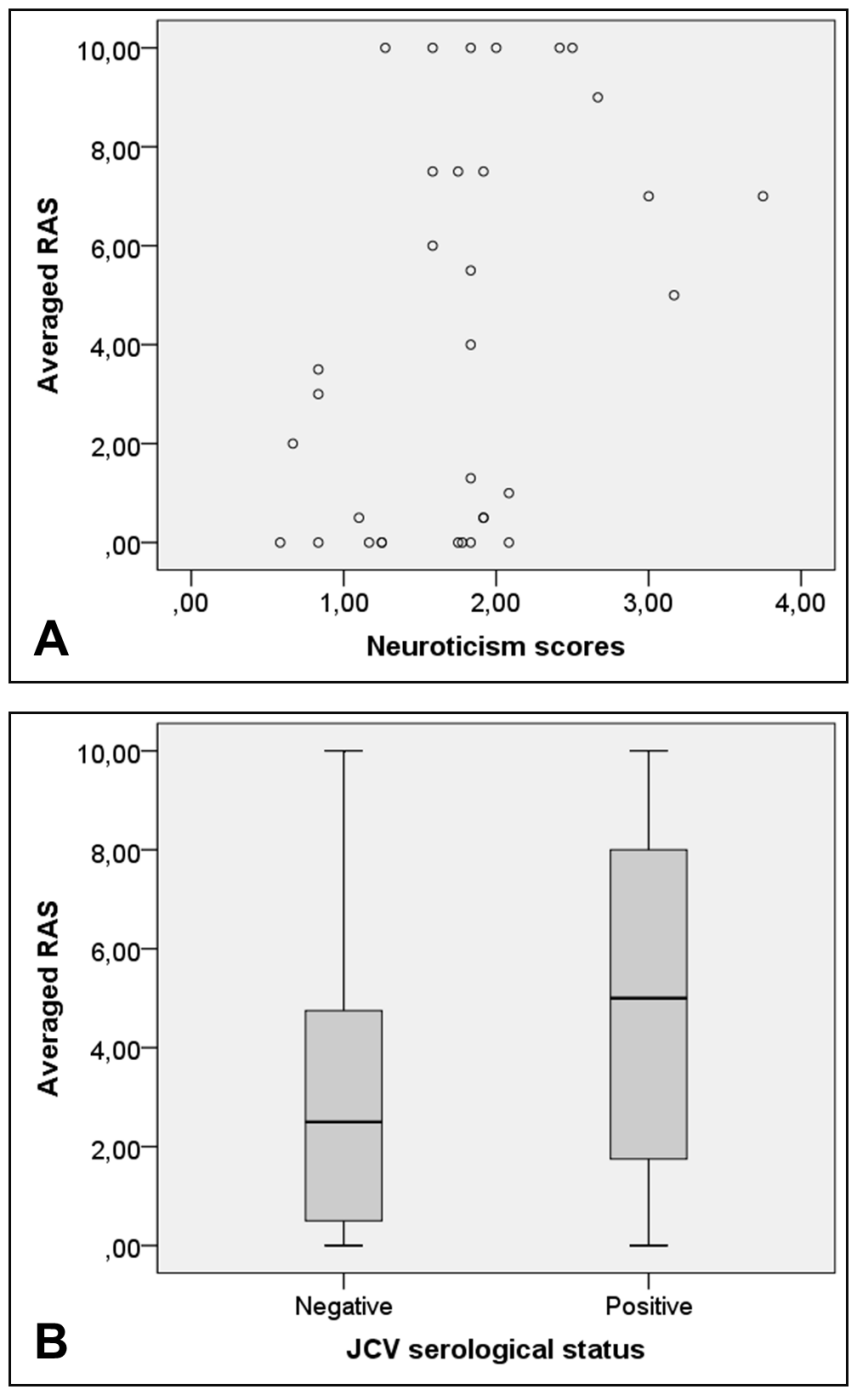
Main factors involved in risk acceptance among NTZ-treated patients. This figure shows the main factors involved in risk acceptance among NTZ-treated patients. **A**. This part shows the positive relationship between neuroticism scores and risk acceptance for high- and very-high-risk therapeutic scenarios among NTZ-treated patients from groups A and B (higher PML risk groups) (p=0.015). **B**. This part shows the relationship between JCV serological status and risk acceptance among NTZ-treated patients from groups C-E (lower PML risk groups): JCV seropositive patients accepted significantly better high- and very-high-risk scenarios (p=0.015).

When only NTZ-treated patients were investigated, only higher neuroticism scores could predict higher ‘averaged RAS’ (p=0.042). When the same analyses were performed in patients from groups A and B, i.e. higher PML-risk patients (considered as a whole), again, only higher neuroticism scores predicted higher ‘averaged RAS’ (p=0.015) (Table **4**; Figure 1 (A)). Instead, when these analyses were performed in patients from groups C to E, i.e. lower PML-risk patients (as a whole), ‘averaged RAS’ were only predicted by the fact of being JCV seropositive (p=0.015). This is, patients who were JCV seropositive had significantly higher ‘averaged RAS’ (Table **4**; Figure 1 (B)).

## Discussion

Over the last years, the investigation into factors involved in the decision-making process in patients with MS as regards new therapeutic approaches has been gaining increasing importance[24]. In fact, as new drugs are developed, it is possible that the prognosis of a non-deniable number of patients, especially those who have an early suboptimal response to first-line DMD[26], depends on their ability to assume risks associated to new treatments. 

In this study, we aimed to investigate the ability of MS patients to accept treatment-associated risks. In particular, we focused on NTZ-treated patients, considering that NTZ is a second-line drug and that these patients may have needed to make a number of decisions as regards their treatments throughout their lives. Despite the unequal numbers of patients within each treatment group, we found that patients on NTZ seemed to be more able to assume higher risks than patients on first-line drugs. This, which was an expected result, also indicates that our survey satisfactorily discriminated between both groups, suggesting that patients’ answers probably reflected patients’ genuine predisposition to accept risks. However, we cannot say with this study whether the differences between NTZ and DMD groups were the result of a selection bias, i.e. since patients currently on NTZ were originally more prone to assuming risks they finally started on NTZ treatment, or whether they were the result of an adaptive process. Namely, it would be possible that NTZ patients’ need for starting on a second-line drug had made them more ‘open’ to assume higher risks. Along these lines, in a study where the authors investigated to what extent MS patients on NTZ treatment were willing to accept increasing hypothetical risks of PML, they reported that patients were more prone to accept higher PML risks than their physicians[24], suggesting that patients on NTZ were more ‘prepared’ to assume higher risks. However, as happened to us, they could not say whether this higher predisposition had appeared before or after being on NTZ treatment. Thus, only longitudinal studies that assess patients’ attitudes towards risky therapeutic options over time will finally tell us whether such possible adaptive process actually exists. 

We also aimed to investigate which factors were influencing risk acceptance in both groups, but especially in the NTZ group. Thus, when all patients were taken as a whole, the only circumstances that marginally determined a higher risk acceptance (apart from the fact of belonging to NTZ or DMD groups) were the perception of the own MS as a more severe disease, and higher neuroticism scores. Interestingly, when only NTZ patients were investigated, the presence of more evident neurotic traits emerged as the only variable significantly associated with a greater predisposition to assume higher risks. The same occurred when only higher PML risk patients were investigated. Whereas it is understandable that a perception of their own MS as a more severe disease may make people more avid to try new drugs, it was somehow unexpected that the perception of MS, in general, as a more severe disease did not have any influence in the amount of risk patients wanted to assume. However, Heesen et al. similarly found that a general perception of MS as a serious disease did not determine the amount of risk the patients considered as acceptable[24], although they did not look at the perception of the disease at an individual level. 

As regards the role of neurotic personality traits, it has been reported that individuals showing higher neuroticism tend to be more worried than others about their future[25]. Moreover, the presence of high neuroticism scores in patients with MS has been associated with higher levels of anxiety and depression[27]. Taking this into consideration, it would have been expected that more neurotic patients had been those more frightened of secondary effects. Instead, we found that those exhibiting more neuroticism were those more prone to assuming higher risks, probably because they were more worried for the disease than for the possibility of secondary effects. This is in agreement with Taillefer et al., who found that, in MS patients, the higher the neuroticism scores were, the more concerned they were about their illness[28]. This would also be in agreement with the finding, in our study, that patients who perceived their own disease as a more severe illness tended to accept higher risks. 

When NTZ patients with lower PML risk were explored, the only variable significantly associated with assuming greater risks was the fact of being JCV seropositive. This is probably the most enthralling result of our study and the best sign in favour of the existence of an adaptive process towards assuming higher risks: patients who knew that were positive, even if they were still on the lower PML risk band, started to assume higher risks earlier than patients who still were JCV seronegative, maybe preparing themselves towards the near future. Interestingly, this adaptive phenomenon would also explain why NTZ patients accepted higher risks than DMD patients in our study. Therefore, considering all these findings, we suggest that risk acceptance is a multifactorial phenomenon, which might be partly explained by this adaptive process and is also intimately related to personality traits. Yet further studies are needed to confirm this hypothesis.

Finally, it must be highlighted the absence of relationship between EDSS scores or ARR and the ability to accept higher risks. This was an unexpected result, since patients with a more aggressive disease, namely patients with higher EDSS scores and ARR, should have probably been more prone to trying new, more risky therapies, if that meant that their chances to slow down their disease activity were greater too. Thus, this result reflexes the complexity of decision-making processes and deserves further investigations. 

Amongst the limitations of the present study there is the cross-sectional design and the relatively low number of DMD-treated patients, meaning that comparisons between treatment groups should be taken with caution, although we were mainly interested in the NTZ group rather than in the comparison between treatment groups. In addition, our patients were not assessed from a cognitive point of view, and the social and educational status was not recorded. Therefore, it would be debatable whether they fully understood the contents presented within the survey or whether their brain pathways involved in planning and decision-making were sufficiently undamaged for us to measure their true ability to make decisions. However, the high internal consistency of patients’ answers in terms of risk acceptance and the highly similar personality traits’ scores between NTZ and DMD groups suggest that patients’ understanding of the content of the survey was correct. Nonetheless, future studies assessing the impact of cognitive dysfunction on risk acceptance and the decision-making process in general are warranted. Another limitation could be the fact that our tool to assess risk acceptance has not been validated yet. Given the lack of standardised tests to assess this point, we designed our own scale, which seems to have accurately measured such treatment-associated risk acceptance, according to the coherence of our results. Further research is nonetheless needed to assess the validity of this test for other studies on MS patients. Finally, we did not study our patients from the imaging point of view and we did not therefore investigate the role of brain damage in risk acceptance abilities. That is, we cannot know whether the presence of a disconnection syndrome secondary to damage in the main white matter tracts[29] might have played a role in our results, as has been suggested for other neuropsychiatric conditions[30]. 

In conclusion, this study suggests that patients on second-line therapies such as NTZ would be more prone to accept higher treatment-associated risks than other MS patients who are on first-line drugs. This, together with the higher risk acceptance amongst JCV seropositive NTZ patients, may denote an adaptive psychological process. On the other hand, the presence of certain personality traits may also determine patients’ willingness to assume higher risks. 

## Supporting Information

Table S1 **In order to help patients to understand the meaning of the therapeutic scenario-associated risks, we also presented five scenarios totally unrelated to MS or MS treatments with similar associated risks.**
(DOCX)
