# Supplementary material for: Risk Acceptance in Multiple Sclerosis Patients on Natalizumab Treatment
Source: PLoS One. 2013 Dec 10;8(12):e82796. doi: 10.1371/journal.pone.0082796 (PMC3858305; doi:10.1371/journal.pone.0082796)
Supplement: Table S1 — In order to help patients to understand the meaning of the therapeutic scenario-associated risks, we also presented five scenarios totally unrelated to MS or MS treatments with similar associated risks. (DOCX) [file pone.0082796.s001.docx]

**Table S1. Hypothetical therapeutic scenarios and non-MS scenarios**

| **Hypothetical therapeutic scenarios with different associated risks** | **Therapeutic scenario-associated risks** | **Non-MS scenarios** |
| --- | --- | --- |
| Very low risk therapeutic scenario | 1/2,000,000 | To die in a plane accident |
| Low risk therapeutic scenario | 1/600,000 | To win the lottery |
| Intermediate risk therapeutic scenario | 1/5,000 | To die in a car accident |
| High risk therapeutic scenario | 1/100 | To be diagnosed with any kind of epilepsy |
| Very high risk therapeutic scenario | 1/50 | To be diagnosed with breast cancer (if you are a woman) |

**Table S1 (footnote).** In order to help patients to understand the meaning of the therapeutic scenario-associated risks, we also presented five scenarios totally unrelated to MS or MS treatments with similar associated risks.
